# Estradiol-17ß Is Influenced by Age, Housing System, and Laying Performance in Genetically Divergent Laying Hens (*Gallus gallus* f.d.)

**DOI:** 10.3389/fphys.2022.954399

**Published:** 2022-07-22

**Authors:** Julia Mehlhorn, Anja Höhne, Ulrich Baulain, Lars Schrader, Steffen Weigend, Stefanie Petow

**Affiliations:** ^1^ Institute for Anatomy I, Medical Faculty and University Hospital, Heinrich-Heine-University Düsseldorf, Düsseldorf, Germany; ^2^ Friedrich-Loeffler-Institut, Institute of Animal Welfare and Animal Husbandry, Celle, Germany; ^3^ Friedrich-Loeffler-Institut, Institute of Farm Animal Genetics, Mariensee, Germany

**Keywords:** estradiol, laying hen, egg laying performance, laying intensity, housing condition, floor housing, cage housing, keel bone damage

## Abstract

The estrogen estradiol-17ß is known as one of the major gonadal steroid hormones with different functions in reproduction. In this study we analyzed estradiol-17ß concentration in laying hens of four pure bred chicken laying lines at four different time intervals of the laying period (17th–19th week of age, 33rd–35th week of age, 49th–51st week of age, and 72nd week of age). The high performing white egg (WLA) and brown egg (BLA) layer lines as well as the low performing white (R11) and brown (L68) layer lines were kept in both single cages and a floor housing system. We investigated whether there were differences in estradiol -17ß concentrations between lines at different ages that could be related to selection for high egg production or phylogenetic origin of the animals, and whether there was an influence of housing conditions on estradiol-17ß. Estradiol-17ß concentrations differed between high and low performing layer lines at all time intervals studied. High performing hens showed higher estradiol-17ß concentrations compared to low performing hens. In all lines, highest estradiol-17ß concentration was measured at their 49th to their 51st week of age, whereas the peak of laying intensity was observed at their 33rd to their 35th week of age. Additionally, hens with fewer opportunities for activity housed in cages showed higher estradiol-17ß concentrations than hens kept in a floor housing system with more movement possibilities. We could show that laying performance is strongly linked with estradiol -17ß concentration. This concentration changes during laying period and is also influenced by the housing system.

## Introduction

Commercial laying hens are continuously bred for high laying performance and have an average laying performance of more than 320 eggs in 13 laying months per hen housed (Preisinger, 2018). At the same time husbandry systems and nutritional requirements of the animal have changed drastically due to high laying performance. Modern laying hens have been bred to have no brood drive, and progressive sexual maturity and shortening of the clutch length interval have been critical factors in significantly improving egg production (Hanlon et al., 2021). To date, little attention has been paid to the underlying physiological changes that made this possible.

The hypothalamic-pituitary-gonadal (HPG) axis plays a central role in controlling reproduction and sexual maturation of animals (Dunn and Sharp, 1990; Bédécarrats et al., 2009). In the HPG axis, Gonadotrophin-releasing hormone (GnRH), a hypothalamic decapeptide, stimulates the secretion of luteinizing hormone (LH) and follicle-stimulating hormone (FSH) in the adenohypophysis. Subsequently, LH regulates the estradiol-17ß synthesis in the ovaries (Clarke and Pompolo, 2005). Through a feedback mechanism, LH and estradiol-17ß inhibit increased GnRH secretion in the brain (Kawashima et al., 1993; Ottinger et al., 2002). As a result, less estradiol-17ß is released. However, modern laying hens have consistently high plasma estradiol-17ß levels (Eusemann et al., 2018a) and it is suggested that this feedback mechanism is disrupted in high performing laying hens or at least changes have occurred in the control of the HPG axis (Hanlon et al., 2021). But the detailed endocrine control mechanism of the laying cycle of the modern laying hen is still little understood, and the feedback between the ovarian follicles and the hypothalamo-hypophysial system which controls follicular development is particularly nebulous.

Estradiol-17ß is the most abundant estrogen and one of the most important gonadal steroids with various functions in the regulation of the female reproduction, e.g., yolk precursor production, oviduct development and reproductive behavior (Yu et al., 1971; Gahr, 2001; Williams et al., 2004). In addition, estradiol-17ß is known to be a triggering factor in calcium and bone metabolism and has a positive effect on bone turnover and regeneration in adults (Bar et al., 1996; Väänänen and Härkötnen, 1996; Johnson, 2000; Beck and Hansen, 2004). Estradiol-17ß is synthesized and permanently produced mainly in granulosa- and theca cells in growing follicles (Marrone and Hertelendy, 1983) with the highest concentrations in small, early-stage follicles and 6–4 h prior the ovulation in laying hens (Johnson and van Tienhoven, 1980; Bahr et al., 1983). During the lifetime of a laying hen, estradiol-17ß concentrations gradually increases until week 20 (onset of laying) and then remain high for the next several weeks (Whitehead and Fleming, 2000; Beck and Hansen, 2004). The exact time course of estradiol-17ß secretion patterns after the peak of production is not clear; only few studies have systematically monitored plasma estradiol over the entire production period. Previous studies have shown that estradiol levels are closely related to the laying performance with estradiol concentrations being highest during the peak of egg production, decreasing during the production year and low during molting (Senior, 1974; Hoshino et al., 1988; Hansen et al., 2003; Ebeid et al., 2008; Habig et al., 2021a). Habig et al. (2021b) also showed differences between different high performing lines during their reproduction cycle. In the pre-laying period (17th week), the estradiol-17ß level of high performing white-egg layers (WLA) was more than twice as high as the level for phylogenetically divergent high performing brown-egg layers (BLA), but much lower than after the onset of laying. In the laying period (25–69 weeks) no significant differences could be observed between the lines, while the estradiol-17ß level continuously increased with age (Habig et al., 2021a; Hanlon et al., 2021).

The influence of estradiol-17ß on egg production was also demonstrated in studies in which egg production was selectively suppressed by the synthetic GnRH agonist deslorelin acetate (Eusemann et al., 2018a). Here, treated hens showed not only lower egg laying performance, but also significantly lower estradiol-17ß levels compared to untreated hens. In addition, there is a correlation between laying performance and estradiol-17ß concentration. Hens of high performing lines achieved higher estradiol-17ß plasma concentrations than hens of low performing lines (Eusemann et al., 2020; Eusemann et al., 2022).

One possible consequence of high laying performance could be the occurrence of keel bone alterations, which are often manifested in reduced bone stability, deviations and fractures. In commercial systems, keel bone damage often affects over 90% of the hens in a flock (Wilkins et al., 2004; Rodenburg et al., 2008; Wilkins et al., 2011; Heerkens et al., 2016). At the onset of sexual maturity, osteoblasts start producing so-called medullary bone. This type of bone is unique to birds (and crocodilians) and serves as a source of calcium for shell formation. At the onset of sexual maturity, osteoblasts begin to form the medullary bone (Whitehead, 2004). Thus, in laying hens, keel bone damage is thought to be related to high laying performance and substantial calcium requirements during eggshell formation (Kerschnitzki et al., 2014). High performing layer lines not only had higher estradiol-17ß concentrations than low performing layer lines, but could also have a significantly higher risk of fracture, a lower degree of mineralization of the cortical bone, and a lower relative amount of medullary bone (Eusemann et al., 2020; Eusemann et al., 2022). The formation of the medullary bone is estrogen and androgen dependent and starts with the onset of ovarian follicle maturation which is part of the hypothalamic-pituitary-gonadal axis (Beck and Hansen, 2004). Therefore, it is likely, that there is a close relationship between laying performance, keel bone damage, and estradiol-17ß concentration.

The influence of different housing conditions of laying hens on the behavior and performance of laying hens in different housing systems has been the subject of numerous studies. Conventional cage systems restrict behavioral expression and increase the risk of skeletal degradation, but floor- or free-range systems evoke difficulties in terms of disease and pest control or higher incidences of skeletal injuries (Whitehead, 2004; Lay et al., 2011; Weeks et al., 2016; Eusemann et al., 2018b). It is obvious that the housing condition have an influence on keel bone damage. The proportion of deviated keel bones was significantly higher in laying hens kept in cages than in floor-housed laying hens whereas fractures occur more often in floor-housed hens (Eusemann et al., 2018b). Additionally, there is a presumption that housing conditions have an influence on laying performance, but the data are inconsistent. At least in enriched and barren cages, egg production seems to be similar (Ylmaz Dikmen et al., 2016; Onbasilar et al., 2020; Philippe et al., 2020) and it is assumed that the differences between the studies are due to the investigated lines and characteristics of the enrichment materials. For free-range hens, Ylmaz Dikmen et al. (2016) described a higher egg production compared to cage housed hens, whereas Philippe et al. (2020) and Shimmura et al. (2010) found lower egg laying rates in hens housed in aviaries or free-range systems compared to cage housed hens. Lower egg laying rates were also found in floor-housed hens compared to cage-housed hens (Voslarova et al., 2006; Ketta et al., 2020). The authors assumed that this could be related to higher animal activity and competition for facilities/resources. Wan et al. (2021) compared two different non-caged systems, namely a plastic-net housing system and a floor-littered housing system and revealed that the plastic-net housing system enhanced the production performance, antioxidant capacity and intestinal health of hens. It should be kept in mind that different findings in different studies could be due to differences in chicken breeds and environmental conditions. Whether the estradiol-17ß concentration is also influenced by the housing system has not yet been investigated.

We hypothesize that activation and function of the HPG axis changed in high performing laying hens to support the significant increase in egg production. Therefore, the aim of the current study was to characterize and compare the estradiol-17ß concentration in pure bred genetically divergent lines with a particular attention to the production cycle and the housing system. We hypothesized that lines with a high ovulation rate would have higher estradiol concentrations compared to low performing lines and that the highest estradiol concentration will be measured at the laying peak. Additionally, we expect that housing condition influences estradiol-17ß concentration, which could explain the lower laying performance in floor-kept hens compared to cage-housed hens as described in previous studies (e.g., Voslarova et al., 2006; Ketta et al., 2020).

## Materials and Methods

### Animals and Housing Conditions

All experiments were performed in accordance with the German Animal Protection Law and were approved by the Lower Saxony State Office for Consumer Protection and Food Safety (No. 33.9-42502-05-10A079).

In this study, we compared phylogenetically divergent high performing white (WLA, *n* = 20) and brown egg laying (BLA, *n* = 20) purebred chicken lines with low performing white (R11, *n* = 20) and brown laying lines (L68, *n* = 20). WLA and BLA originated as purebred lines from the breeding program of Lohmann Breeders and have been kept at the Institute of Farm Animal Genetics of the Friedrich-Loeffler-Institute since 2012. L68, and R11 are very old laying lines that have been maintained as conservation lines at the institute for decades. This four-line animal model was developed as part of a multidisciplinary collaboration at the Friedrich-Loeffler-Institute and first presented by Lieboldt et al. (2015), who described the growth and performance of the four chicken lines. Since then, a number of studies have been conducted, including bone traits (Habig et al., 2017; Dudde et al., 2020) and keel bone damage (Eusemann et al., 2018a; Eusemann et al., 2020; Habig et al., 2021a).

All animals hatched at the same day and were raised separately in a floor housing system until 16 weeks of age. Rearing compartments (6 m × 4 m) were littered with wood-shavings and straw and were equipped with perches. Food (week 1–7: 12.97 MJ AMEN/kg DM, 189.61 g/kg crude protein, 31.38 g/kg crude fat, 9.14 g/kg Ca, 6.94 g/kg P; week 8–16: 12.82 MJ AMEN/kg DM, 151.67 g/kg crude protein, 30.21 g/kg crude fat, 15.83 g/kg Ca, 8.11 g/kg P) and water was provided *ad libitum*. A standard light-programme was applied during rearing period. At 16 weeks of age 10 hens of each line were moved to a single cage housing system equipped with a food trough, two drinking nipples and a perch. Other 10 hens of each line were kept in floor pens (each 2.0 m × 4.0 m) separated by line. The animals were distributed between two pens per line. Both housing conditions were in the same room. Floor pens were littered with wood-shavings and equipped with perches and nests mounted on a slatted floor 0.5 m above the litter area. In both housing systems animals had *ad libitum* access to food (11.68 MJ AMEN/kg DM, 168.11 g/kg crude protein, 29.43 g/kg crude fat, 50.05 g/kg Ca, 5.06 g/kg P) and water. The light period increased from 9 to 14 h from 16th until 20th week of age and was maintained at 14 h for the remainder of the laying cycle (to 72 weeks of age).

Laying performance was recorded individually of all hens in the single cages and summarized for every week. However, recording laying performance in the floor-housed hens at individual level was not possible, therefore, we determined the total number of eggs per week and chicken line. For analyses, we calculated the laying intensity (in %) based on the number of eggs laid during the experimental weeks of age and the number of hen days (number of hens x number of days).

We investigated four time periods, the first period from the 17th, 18th, and 19th week of age (before start of laying), the second period from the 33rd, 34th, and 35th week of age (maximum of egg production), the third period from the 49th, 50th, and 51st week of age (decrease in egg production in low producing lines) and the last period in the 72nd week of age (end of experiment).

### Blood Sampling

In each of the four experimental periods blood was collected once a week between 2.00 and 5.00 p.m. For this, ten hens of each line and housing condition were selected (resulting in a total of 80 hens). Blood was collected from the wing vein (*V. ulnaris*) using needles with a gauge of 21 and 2 ml syringes (both Henry Schein, Hamburg, Germany). After collection, blood samples were immediately transferred to K_3_-EDTA covered tubes (Greiner, Solingen, Germany) to avoid coagulation, and centrifuged at 4°C for 10 min at 2750 rcf. Tubes were placed on ice, and plasma phase was pipetted into 1.5 ml Eppendorf^®^ cups (Fisher Scientific, Schwerte, Germany) and frozen at −20°C until analysis.

### Hormone Assays

For estradiol-17ß analysis a commercial estradiol-17ß ELISA Kit (IBL International, Hamburg, Germany) was used. Assays were conducted following the manufacturer’s instructions and samples were analyzed in duplicates. The kit used detects the entire E2. The absorption of the plate was detected with an ELISA microplate reader (Tecan, Crailsheim, Germany) at 450 nm with a reference wavelength at 620 nm.

The concentrations and coefficients of variation of the assays were calculated using the microplate reader software (Magellan^®^ Version 7.1, Tecan Austria, Salzburg, Austria). For estradiol the intraassay coefficient of variation was 6% and the interassay coefficient of variation was 9%.

### Statistical Analysis

Data were statistically analyzed using JMP, version 15.0 (Statistical Analysis System Institute, Cary, NC, United States). Estradiol-17ß content was examined using a linear mixed model. Individual hen was included as random factor in order to account for repeated measurements within animal. Layer line, housing condition, age and the three two-way interactions were considered as fixed effects. The 3-fold interaction between housing condition, line and age did not show a significant effect on the Estradiol concentration, therefore it was removed from the model (model 1).

Laying performance of single caged hens was analyzed using a linear mixed model, too, including individual hen as random effect. Layer line, age and the interaction of layer line x age were considered as fixed effects (model 2). The influence of layer line and age on group laying performance of floor housed hens (two pens per line) was analyzed using a two-way ANOVA including layer line and age with interaction (model 3).

Differences between Least Squares means (LSM) were tested by means of the Tukey-Kramer Test, adjusting for multiple comparisons. Differences were regarded as statistically significant at *p* < 0.05.

## Results

From Table 1 it is evident that estradiol-17ß plasma concentration was significantly affected by housing condition, layer line, age and by the interaction of line*age (*p* < 0.05). The laying performance, analyzed separately by housing condition, was significantly influenced by layer line, age and also by the line*age interaction (*p* < 0.001).

**TABLE 1:** Significance of fixed effects (*p*-values).

Source	Estradiol-17ß ^a^	Laying intensity ^b^	Laying intensity ^c^
		Cage housing	Floor housing
Housing condition (HC)	**0.0019**	—	—
Layer line (LL)	**<0.0001**	**<0.0001**	**<0.0001**
Week of age	**<0.0001**	**<0.0001**	**<0.0001**
HC * LL	0.8415	—	—
HC * week of age	0.1113	—	—
LL * week of age	**<0.0001**	**0.0001**	**<0.0001**
HC * LL * week of age	0.2422	—	—

a Model (1).

b Model (2).

c Model (3).

### Estradiol-17ß Concentration in Different Layer Lines

**FIGURE 1:**
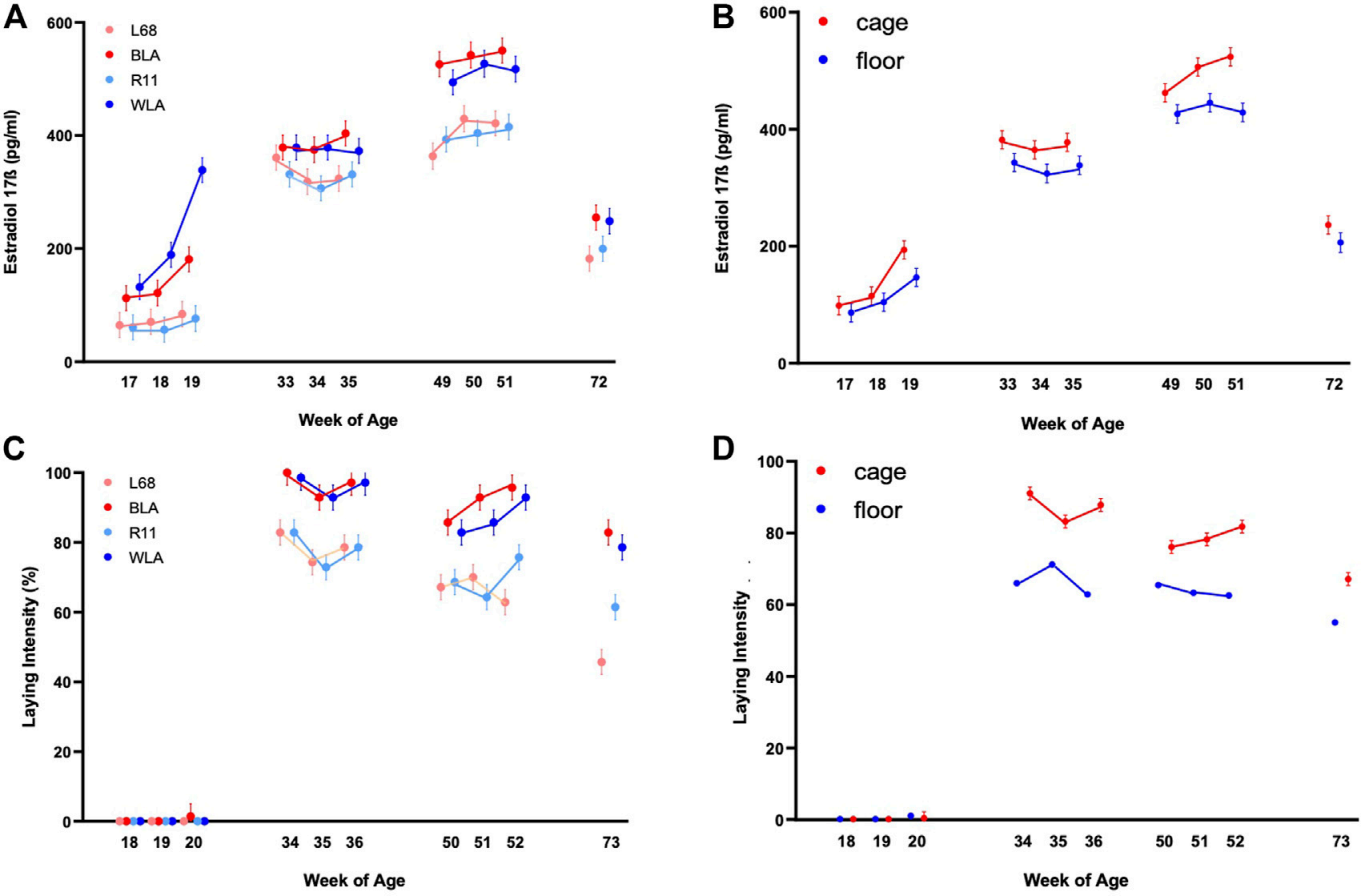
Estradiol-17ß blood plasma concentration (pg/ml) and laying intensity (%) of four different layer lines kept in different housing systems and investigated at four time periods (17th, 18th, and 19th week of age (before start of laying); 33rd, 34th, and 35th week of age (maximum of egg production); 49th, 50th, and 51st week of age (decrease in egg production in low producing lines) and 72nd week of age (end of experiment). The laying intensity is shown after each week of blood sampling **(A)** Estradiol-17ß concentration of the two high performing layer lines (WLA, BLA) and the two low performing layer lines (L68, R11) respectively cage-housed and floor-housed laying hens **(B)**, consisting of WLA, BLA, L68, and R11 hens) during their laying period **(C)** Laying intensity of all four-layer lines and of cage-housed and floor-housed laying hens **(D)** during their laying period.

High performing laying hens (BLA, WLA) started in week 17 with estradiol-17ß plasma concentrations almost twice as high as low performing laying hens (L68, R11) and showed a higher increase from week 18 to week 19 compared to the low performance lines (Figure 1A; Supplementary Table S1). In the period of maximum laying performance (weeks 33–35) the high performing lines showed a tendency towards higher estradiol-17ß concentrations than those of low performing lines.

In all lines, estradiol-17ß concentration increased until week 50 or 51 (3rd period), reaching more than 500 pg/ml in high performing hens (WLA: 527.03 ± 22.04 pg/ml, BLA: 550.42 ± 22.01 pg/ml) and about 400 pg/ml in low performing hens (L68: 429.58 ± 22.54 pg/ml, R11: 415.21 ± 22.04 pg/ml). After week 51, estradiol-17ß concentration decreased to 248.58 ± 23.59 pg/ml in WLA, 255.00 ± 22.64 pg/ml in BLA, 199.50 ± 22.54 pg/ml in R11 and 182.00 ± 23.14 pg/ml in L68 in week 72.

### Estradiol-17ß Concentration in Different Housing Systems

There is a significant main effect of the housing system on estradiol-17ß concentration in laying hens (Figure 1B, Supplementary Table S1). In all study periods, caged hens tended to have higher estradiol-17ß concentrations than floor-housed hens, with the difference being statistically significant only at 51 weeks of life. (Figure 1B; Supplementary Table S2). Within both housing systems, estradiol-17ß concentrations differed significantly between all the 4 periods. In both, floor pens and cages, estradiol-17ß concentrations increased until the 3^rd^ period and were highest between week 49 and 51 (floor: 445.16 ± 15.76 ng/ml, cage: 523.81 ± 15.57 ng/ml). In week 72, estradiol-17ß concentration decreased to 206.24 ± 16.89 ng/ml in floor-housed hens and 236.29 ± 15.58 ng/ml in cage-housed hens, respectively.

### Laying Intensity

Laying intensity of cage housed hens was significantly affected by the interaction between week of age and breeding line (*p* < 0.0001); Supplementary Table S3 and Figure 1C show that within the first period hens had not yet started laying. In the second period all lines reached the maximum egg laying rate in the 34th week of life (BLA: 100.00% ± 3.60, WLA: 98.57% ± 3.60, L68, and R11: 82.86 ± 3.60). In all periods high performing layers showed a higher laying intensity compared to low performing layers, with statistically significances in the 2nd, 3rd, and 4th period. Since the laying performance of hens kept in floors was not recorded individually, we only note descriptively that hens kept in cages had a higher laying intensity than hens kept in floor systems in time periods 2, 3, and 4 (Figure 1D; Supplementary Table S3), without this being statistically verifiable.

## Discussion

Genetic selection for earlier sexual maturation and extended production cycles in laying hens has significantly improved reproductive efficiency (Hanlon et al., 2021). and allowed modern layers to double their reproductive capacity compared to their 1960s counterparts. Breeding improvement in egg production has led to a continuous improvement in laying performance, with a trend of an increase of about 2–3 eggs per hen per year in a 13-months production cycle (Preisinger, 2018) However, the underlying physiological changes throughout the laying period and the correlation between these changes and laying performance have received limited attention. Hence, the effect of housing conditions, genotype, and laying performance and their interactions on estradiol concentration is not fully understood.

In this study, we investigated the relationship between blood estradiol-17ß levels and egg production at four different time points during the laying period in two high performing laying lines (WLA and BLA) and two low performing laying lines (L68 and R11). All lines showed low estradiol-17ß concentrations in the beginning of the 1st investigated time period (17th week of age), followed by a strong increase in the 2nd period (33rd–35th week of age) and an even higher estradiol-17ß concentration in the 3rd period (49th–51st week of age). In the 4th period (72nd week of age) estradiol-17ß concentration decreased to a level between the first and 2nd period. Low estradiol-17ß concentrations at the beginning of the first period are not unexpected because at this time, hens are just starting to lay and are not yet sexually matured. WLA and BLA not only showed higher estradiol-17ß concentrations but also a higher increase from week 18 to week 19 compared to the low performance lines.

Our finding that there are differences in estradiol-17ß concentrations between WLA and BLA in the pre-laying period is in line with Habig et al. (2021b). Probably, these differences are due to the different phylogenetic background of these lines.

Interestingly, estradiol-17ß concentration in the second period is high, but it is still exceeded by the concentration of the third period. Due to its involvement in the regulation of egg production (Yu et al., 1971; Williams et al., 2004) we expected highest estradiol concentrations at the maximum of egg laying rather than in the following period. Next to its function in the female reproduction cycle, estradiol-17ß is also involved in calcium and bone metabolism and has an impact on bone stability (Bar et al., 1996; Väänänen and Härkötnen, 1996; Johnson, 2000; Beck and Hansen, 2004). Previous studies have shown that most keel bone fractures occur or were already present at this week of age, when we measured the highest estradiol-17ß concentrations (Petrik et al., 2015; Stratmann et al., 2015; Eusemann et al., 2018b). Estradiol-17ß can also counteract the reduction of bone strength and loss of structural bone as a consequence of increased egg production (Whitehead and Fleming, 2000). The medullary bone, which presents a calcium reservoir for egg shell building, is formed at the onset of egg laying (Senior, 1974; Johnson, 2000; Whitehead and Fleming, 2000; Dojana et al., 2015). The key role of Estradiol-17ß in the modelling of the medullary bone is well described (e.g., Hiyama et al., 2012; Squire et al., 2017; Eusemann et al., 2022) Thus, our results may support the relationship between estradiol-17ß and bone health. In the investigation of Eusemann et al. (2020) the authors could show, that hormonally castrated hens have significantly less fractures than the non-castrated control group. The increase of estradiol-17ß after the peak of egg production in our study might be related with its function in regeneration in adult bone turnover particularly in the high performing selected lines. Interestingly, this is different to non-poultry birds like starlings. Here, estradiol-17ß concentrations decrease after onset of laying well before the final yolky follicle was ovulated (Williams et al., 2004). Apparently, ovary and oviduct size and their function are maintained despite lower estradiol-17ß concentration. The authors suggest that one reason for this might be that there are negative, non-reproductive effects of high estradiol-17ß levels which would foster rapid down-regulation, e.g., suppression of hematopoiesis or immunosuppression, or decreased embryo viability. Alternatively, the observed decrease in estradiol-17ß concentration in starlings could be a pre-requisite for rapid oviduct regression at the end of laying, because estrogens also oppose the induction of apoptosis that is involved in oviduct regression (Williams and Ames, 2004). It is not surprising, that such a decrease would be not observed in laying hens, or at least only at a much later point of reproduction cycle, because of their extended laying period and extended end of reproduction period.

Estradiol-17ß concentration in cage-housed hens was higher compared to floor-housed hens where birds had more opportunities for activity. Aguado et al. (2015) described a reduced bone mass and bone quality in hypoactive chickens. There are two possible explanations for this: either, the stimulating effect of activity/exercises on bone strength is related to the degree of biomechanical load experienced by the bone [following the concept of causal histogenesis (Pauwels, 1960)] or it is caused by a lower estradiol-17ß concentration. Although we cannot state whether the higher estradiol-17ß concentration in cage-housed hens is the consequence or the cause of less activity, it seems likely that there is a correlation between these two parameters. Besides, the estradiol-17ß concentration depends on the number of maturing follicles. Thus, the lower laying intensity may also be an explanation for the lower estradiol-17ß concentration in floor-housed hens compared to cage-housed hens. Recent studies showed that, at least in premenopausal women, high physical activity was associated with lower levels of estrone and estradiol (Matthews et al., 2012; Dallal et al., 2016). These findings suggest that physical activity may induce changes in estrogen metabolism possibly through more extensive hydroxylation of parent estrogens, leading to increased excretion. Given the exploratory nature of these studies, findings should be interpreted cautiously, but in our opinion, there could be a negative relationship between activity and estradiol-17ß level in the blood as floor-housed laying hens are more active/more mobile than cage housed hens.

The laying performance of the lines used in our study was significantly affected by the genotype which agrees with previous studies (e.g., Lieboldt et al., 2015). WLA and BLA were classified as high performing lines with a laying intensity of more than 93%–95% at the maximum of egg production and when housed in cages, whereas cage-housed low performing L68 and R11 showed a lower egg production of 67%–74%. Besides, laying maturity, defined as age at the first egg laid, was reached by the hens of the high performing genotypes four to 5 weeks earlier than in hens of the low performing genotypes, namely in the 20th week of age (Lieboldt et al., 2015). In our study we did not investigate the exact age of reaching laying maturity but observed that laying intensity in week 20 was still 0% in WLA, L68, and R11 and just 1.4% in BLA. Thus, reaching laying maturity must be a little bit later in our study. However, laying intensity of all four lines was even a little bit higher as shown in Lieboldt et al. (2015) and all four lines reached their maximum egg production in the 34th week of age.

Determining laying performance in floor-housed hens is always a challenge. Some eggs might be laid in the litter and were not easily counted, an assignment to the individual hen is difficult and it is possible that eggs were completely destroyed and the remains buried in the litter. Another problem could be that there might be hens among the floor housed hens, that have not laid and therefore have low estradiol-17ß levels. In order to exclude the possibility that these animals decrease the estradiol-17ß level of the whole group, we checked it and would have excluded all hens with values with the level of the 1st period. But this was not the case, apparently all floor-housed hens always have laid regularly.

The observation that high performing lines showed higher concentrations of estradiol-17ß compared to both low performing layer lines, independent of the phylogenetic origin, supports our hypothesis that selection for egg production resulted in higher concentrations of estradiol-17ß. It seems that high estradiol-17ß concentrations are related to production level and might be a result of human directed selection. Hanlon et al. (2021) hypothesized that modifications in the control of the hypothalamic-pituitary gonadal axis have occurred in modern laying hens. They compared estradiol-17ß concentration and mRNA levels of key genes involved in the HPG axis of current commercial hens (Lohmann LSL-lite) with Shaver White leghorns as 2000s commercial equivalents and Smoky Joe hens as 1960s commercial equivalents. Their results showed that the extended laying persistency in Lohmann LSL lite hens was supported by sustained pituitary sensitivity to GnRH and recurrent increases in follicle-stimulating hormone (FSH) and estradiol-17ß. This is in line with our finding about higher estradiol concentration in high performing layers compared to low performing layers. In Hanlon et al. (2021) the highest estradiol-17ß concentrations were found (in all strains) at the beginning of laying at week 19–21. Up to 100th week of age, there are recurrent elevations of estradiol concentration, but they do not reach the level of the first peak. This does not agree with our results where there was just one peak of estradiol-17ß concentration and much later at week 50–51. However, these differences could be caused by the different genotypes of the used lines or differences in e.g. feeding, light regime or rearing. Besides, blood sampling in Hanlon et al. (2021) was taken in the morning and not in the afternoon as in our study what makes a direct comparison difficult. It is known, that there are circadian fluctuations in estradiol-17ß concentrations since the highest concentrations of estradiol-17ß was observed 6–4 h prior the ovulation in laying hens (Johnson and van Tienhoven, 1980; Bahr et al., 1983). In the course of the daily egg laying cycle blood concentrations clearly reflected the stage of egg formation (Habig et al., 2021a). The egg-laying cycle of modern laying hens lasts about 24 h and oviposition followed 24 h after ovulation (Bain et al., 2016). As modern layer lines lay their eggs all about the same time (more likely in the morning), ovulation and peak of estradiol-17ß concentration should be as well at the same time. Thus, it cannot be excluded that estradiol-17ß concentrations measured in the afternoon differ from estradiol-17ß concentrations measured in the morning. However, Habig et al. (2021b) also showed that the daily variations of the estradiol-17ß concentrations were rather small compared to differences between different ages or the phylogenetic origin of chicken lines.

Habig et al. (2021b) showed differences between the estradiol-17ß level of WLA compared to BLA in the pre-laying period (17th week) and assumed that this might be indication that brown-egg layers were further from sexual maturity than white-egg layers (although start of egg laying was similar). In our study we found the same results but shifted to the 19th week of age. Here, the WLA hens were as twice as high as the BLA hens in their estradiol-17ß concentrations.

As modern laying hens further selected for improved laying performance, the physiological implications of this intensive selection must be considered. Here, many factors play a role but are not fully described for the laying hen. There are particularly open questions regarding the physiological and neuronally controlled mechanisms for a high laying performance and there are still ambiguities about the influence of the housing system. In addition to pituitary-derived gonadotropins, which are essential for steroid production, chicken ovarian steroidogenesis is under regulatory influence of several endocrine, paracrine and autocrine factors (Sechman, 2013), thus, further investigations are necessary to investigate all parameters which are involved in reproductivity over the whole laying period.

## Data Availability

The original contributions presented in the study are included in the article/Supplementary Material, further inquiries can be directed to the corresponding authors.
