# Supplementary material for: Estradiol-17ß Is Influenced by Age, Housing System, and Laying Performance in Genetically Divergent Laying Hens (Gallus gallus f.d.)
Source: Front Physiol. 2022 Jul 22;13:954399. doi: 10.3389/fphys.2022.954399 (PMC9353941; doi:10.3389/fphys.2022.954399)
Supplement: Supplementary file 1 [file Table1.docx]

**Supplementary Material**

Table S1. Measurements of estradiol-17ß (pg/ml) in laying hens of four lines at different weeks of age (model 1, LS-Means ± SE)

| Week of age | Layer line | | | |
| --- | --- | --- | --- | --- |
|  | L68 | BLA | R11 | WLA |
| 17 | 64.9 ± 22.04^a^ | 112.3 ± 22.01^a^ | 60.9 ± 22.04^a^ | 132.0 ± 22.53^a^ |
| 18 | 70.7 ± 22.04^ab^ | 121.6 ± 22.01^ab^ | 56.7 ± 22.04^b^ | 189.2 ± 22.04^a^ |
| 19 | 84.4 ± 22.04^b^ | 181.2 ± 22.01^b^ | 76.6 ± 22.04^b^ | 339.0 ± 22.04^a^ |
| 33 | 360.9 ± 22.04^a^ | 378.7 ± 22.01^a^ | 331.3 ± 22.04^a^ | 378.9 ± 22.04^a^ |
| 34 | 318.3 ± 22.04^a^ | 374.9 ± 22.01^a^ | 306.7 ± 22.54^a^ | 378.6 ± 22.04^a^ |
| 35 | 324.0 ± 22.04^a^ | 403.7 ± 22.01^a^ | 331.1 ± 22.54^a^ | 372.8 ± 22.04^a^ |
| 49 | 363.6 ± 22.54^b^ | 526.2 ± 22.50^a^ | 393.2 ± 22.04^b^ | 494.2 ± 22.04^ab^ |
| 50 | 429.6 ± 22.54^ab^ | 542.4 ± 22.01^a^ | 404.3 ± 22.04^b^ | 527.0 ± 22.04^a^ |
| 51 | 421.6 ± 23.14^b^ | 550.4 ± 22.01^a^ | 415.2 ± 22.04^b^ | 517.6 ± 22.04^ab^ |
| 72 | 182.0 ± 23.14^a^ | 255.0 ± 22.64^a^ | 199.5 ± 22.54^a^ | 248.6 ± 23.59^a^ |

a,b means within row with no common letter differ significantly (p < 0.05)

^a^ for details see Material and Methods

Table S2. Measurements of estradiol-17ß (pg/ml) in laying hens in different housing systems and different weeks of age (model 1, LS-Means ± SE)

| Week of age | Housing | |
| --- | --- | --- |
|  | Floor | Cage |
| 17 | 86.5 ± 15.76^a^ | 98.5 ± 15.57^a^ |
| 18 | 104.3 ± 15.59^a^ | 114.8 ± 15.57^a^ |
| 19 | 146.8 ± 15.59^a^ | 193.8 ± 15.57^a^ |
| 33 | 343.1 ± 15.59^a^ | 381.8 ± 15.57^a^ |
| 34 | 324.3 ± 15.76^a^ | 364.8 ± 15.57^a^ |
| 35 | 338.2 ± 15.76^a^ | 377.6 ± 15.57^a^ |
| 49 | 426.3 ± 15.76^a^ | 462.3 ± 15.75^a^ |
| 50 | 445.2 ± 15.76^a^ | 506.5 ± 15.57^a^ |
| 51 | 428.6 ± 15.98^b^ | 523.8 ± 15.57^a^ |
| 72 | 206.2 ± 16.89^a^ | 236.3 ± 15.58^a^ |

a,b means within row with no common letter differ significantly (p < 0.05)

Table S3. Laying intensity (%) of layer lines at different weeks of age, separately analysed in cages and floor housing systems (model 2, LS-Means ± SE, model 3)

| Housing | Week of age^1^ | Layer line | | | |
| --- | --- | --- | --- | --- | --- |
|  |  | L68 | BLA | R11 | WLA |
| Cage | 20 | 0.0 ± 3.60^a^ | 1.4 ± 3.60^a^ | 0.0 ± 3.60^a^ | 0.0 ± 3.60^a^ |
|  | 34 | 82.9 ± 3.60^a^ | 100.0 ± 3.60^a^ | 82.9 ± 3.60^a^ | 98.6 ± 3.60^a^ |
|  | 35 | 74.3 ± 3.6^ab^ | 92.9 ± 3.60^a^ | 72.9 ± 3.60^b^ | 92.9 ± 3.60^a^ |
|  | 36 | 78.6 ± 3.60^a^ | 97.1 ± 3.60^a^ | 78.6 ± 3.60^a^ | 97.1 ± 3.60^a^ |
|  | 50 | 67.1 ± 3.60^b^ | 85.7 ± 3.60^a^ | 68.6 ± 3.60^b^ | 82.9 ± 3.60^a^ |
|  | 51 | 70.0 ± 3.60^b^ | 92.9 ± 3.60^a^ | 64.3 ± 3.60^b^ | 85.7 ± 3.60^a^ |
|  | 52 | 62.9 ± 3.60^b^ | 95.7 ± 3.60^a^ | 75.7 ± 3.60^b^ | 92.9 ± 3.60^a^ |
|  | 77 | 45.7 ± 3.60^b^ | 82.9 ± 3.60^a^ | 61.4 ± 3.60^b^ | 78.6 ± 3.60^a^ |
|  |  |  |  |  |  |
|  |  |  |  |  |  |
| Floor^2^ | 20 | 0 | 0 | 0 | 0 |
|  | 34 | 54.8 | 85.7 | 36.8 | 86.6 |
|  | 35 | 61.7 | 89.1 | 39.7 | 94.4 |
|  | 36 | 50.3 | 81.9 | 28.6 | 90.6 |
|  | 50 | 42.4 | 73.3 | 51.9 | 94.2 |
|  | 51 | 41.7 | 74.3 | 46.1 | 91.2 |
|  | 52 | 35.3 | 80.5 | 47.5 | 87.0 |
|  | 73 | 28.2 | 66.7 | 41.6 | 83.6 |

a,b means within row with no common letter differ significantly (p < 0.05)

1. The laying performance is shown after each week of blood sampling
2. Laying intensity in floor housing is based on group means
